# Mechanism of Zn alleviates Cd toxicity in mangrove plants (*Kandelia obovata*)

**DOI:** 10.3389/fpls.2022.1035836

**Published:** 2023-02-02

**Authors:** Shan Chen

**Affiliations:** Third Institute of Oceanography, Ministry of Natural Resources, Xiamen, China

**Keywords:** zinc, cadmium, *Kandelia obovata*, phenolic acid metabolism, reactive oxygen species

## Abstract

Cadmium (Cd) pollution is very common and serious in mangrove ecosystems in China. Zinc (Zn) has been used to reduce Cd accumulation in plants, and phenolic acid metabolism plays an important role in plant response to stress. In present study, in order to clarify whether Zn alleviates Cd toxicity in mangrove plants through phenolic acid metabolism, the Cd-contaminated *Kandelia obovata* plants were treated with different concentrations of (0, 80,300, and 400 mg·kg^–1^) ZnSO_4_ in a set of pot experiments and the biomass, the contents of Cd, Zn, soluble sugar, chlorophyll and the activities of 1,1-diphenyl-2-picrylhydrazyl (DPPH), ferric-reducing antioxidant power (FRAP), l-phenylalanine ammonia-lyase (PAL), shikimic acid dehydrogenase (SKDH), cinnamyl alcohol dehydrogenase (CAD) and polyphenol oxidase (PPO) in the leaves were analyzed. The results showed that Cd contents in the leaves of *Kandelia obovata* ranged from 0.077 to 0.197 mg·kg^–1^ under different treatments, and Zn contents ranged from 90.260 to 114.447 mg·kg^–1^. Low-dose ZnSO_4_ treatment (80 mg·kg^–1^) performed significant positive effects on the biomass, phenolic acid metabolism-related enzyme activities, antioxidant capacity, and chlorophyll and soluble sugar contents in the leaves of Cd-contaminated mangrove plants. At the meantime, the addition of low-dose ZnSO_4_ promoted the biosynthesis of hydroxycinnamic acid, hydroxybenzoic acid, and enhanced the plant antioxidant capacity, thus alleviated Cd toxicity in mangrove plants.

## Introduction

Mangroves are located in the interlaced zone of coastal areas in tropical regions, and constitute a high-yield ecosystem that supports a variety of plants and animals through the food chain (Bharathkumar et al., 2007). Some common and widely distributed mangrove species, such as *Aegiceras corniculatum*, *Sonneratia caseolaris* and *Kandelia obovata*, have useful metabolite extracts with great medicinal value (Chen et al., 2012). The economic value of mangroves brings much wealth to human beings; however, various interferences of human activities, such as mining wastes, metal smelting waste residues, and untreated domestic sewage causes mangroves to suffer from serious heavy metal pollution, and their habitats are decreasing in size (Das et al., 2016). Previous research showed that mangroves suffer from serious cadmium (Cd) and zinc (Zn) pollution, and are tolerant to various metal pollution in their environment (Sundaramanickam et al., 2016; Analuddin et al., 2017). Cd is a widely existing nonessential element that is classified as a harmful heavy metal to human health (Chao et al., 2009; Shang et al., 2020). Cd can be absorbed and accumulated by some plants and flows into higher nutrient level organisms through the intricate food chain (Adamczyk-Szabela et al., 2020), threatening human health (Bodin et al., 2013; Chen et al., 2021). Zn is a familiar essential element of plants and animals, and plays a fundamental role in stabilizing and protecting biofilms from oxidative and peroxidative damage (Zhang et al., 2022). However, high levels of Zn can also cause heavy metal pollution in mangrove ecosystems, leading to restricted plant germination, reduced root development and induced plant aging (Lefevre et al., 2014; Chen et al., 2019). Cd and Zn are in the same group with similar physical and chemical properties and always exist together in nature (Zha et al., 2004; Mongkhonsin et al., 2016). Mongkhonsin et al. (2016) proved that Zn can reduce Cd toxicity under Cd and Zn treatments in *Gynura pseudochina*. Our previous studies also found that 100 mg·kg^–1^ Zn treatment can ease Cd toxicity in *K. obovata* (Chen et al., 2019). Many researchers have speculated that this phenomenon is caused by phenolic acid metabolism, but no one has specified what the mechanism is or tested any hypothesis.

Phenolic compounds are secondary metabolites consisting of hydroxylated aromatic compounds and carbon-based compounds, which are only found in plants and microorganisms (Tato et al., 2013). Phenolic compounds can protect plant tissues from wounds, oxidative damage, insects and pathogen infections (Ali et al., 2005). For example, *K. obovata* contains various common phenols, such as cinnamic acids, flavonoids and phenylpropanoid derivatives, and their ecological functions have been tested *in vitro* antioxidant and heavy metal bioavailability assays (Lu et al., 2007; Li et al., 2016; Jiang et al., 2017). Phenolic compounds play a variety of important chemical and biological functions in plants to adapt to various changing environments. These physiological processes are metabolic plasticity because plants can respond to external pressures by rapidly inducing phenolic compound synthesis in a reversible way (Tanveer et al., 2022). For instance, the addition of Cd significantly increased the total content of phenol in mangrove species such as *K. obovata* (Kováčik et al., 2009). Numerous studies have also reported that the increase in phenolic compounds in plant tissues and root secretions is a special response to different biological and abiotic stresses (Tato et al., 2013). ZnSO_4_-treated *K. obovata* plant showed higher biomass and stronger antioxidative capacity as compared to only CdCl_2_ treated *K. obovata* plant due to the enhancement of its phenolic biosynthesis (Zhao and Zheng, 2015; Chen et al., 2019). Phenolic acids are mainly synthesized by the shikimic acid and phenylpropanoid pathways (Ali et al., 2005; Barros et al., 2019). The precursors of shikimic acid-mediated phenolic acid synthesis are mainly aromatic amino acids, phenylpropyl amino acids, and tryptophan produced by simple carbohydrate glycolysis and the pentose phosphate pathway (Abdulrazzak et al., 2006). The shikimic acid pathway is a common pathway that provides precursors for subsequent secondary metabolites. It also shows how primary and secondary aromatic metabolism are related. It has been estimated that 60% of the total plant biomass consists of molecules passing through the shikimic acid pathway (Tato et al., 2013). According to the above analysis, the soluble sugar content in plant leaves directly influences the amount of phenolic compound synthesis, and the synthesis of soluble sugar is formed by the photosynthesis of plants, while plant photosynthetic ability is closely related to leaf chlorophyll content. Therefore, this experiment measured the soluble sugar content and chlorophyll content of plants to assess their phenolic acid metabolism. Phenolic compound metabolism-related enzymes include l-phenylalanine ammonia-lyase (PAL), which can catalyze phenylalanine to cinnamate; shikimic acid dehydrogenase (SKDH), which can provide substrate for PAL; cinnamyl alcohol dehydrogenase (CAD), which can provide precursors for the synthesis of lignin; and polyphenol oxidase (PPO), which can catalyze the oxidation of catechol to catechol diquinone and act on the substrate monophenol monooxygenase (Kováčik et al., 2009). These phenolic acids are considered to be effective substances protecting plants against oxidative damage caused by heavy metal stress. The structure of phenolic acids endows them with a strong ability to scavenge free radicals and chelate heavy metals, which prevents Fenton reactions. In particular, phenolic acids such as caffeic acid, chlorogenic acid, ferulic acid, and *p*-coumaric acid have been shown to have greater antioxidant capacity than hydroxyl derivatives of benzoic acid such as *p*-hydroxybenzoic acid, vanillic acid, and syringic acid (Sgherri et al., 2003). Phenolic acids not only function in free radical scavenging but also inhibits lipid peroxidation and electron donors (Shi et al., 2010; Maqsood et al., 2014). Therefore, they can be used as excellent reaction substrates for some antioxidant enzymes (peroxidases) to reduce oxidative stress (Oh et al., 2009; Posmyk et al., 2009). In addition, phenolic acids can protect photosynthetic organs from light damage under heavy metal stress (Burchard and Weissenbock, 2000; MacFarlane and Burchett, 2000). Recently, it has been reported that phenolic acid content is related to the heavy metal tolerance process of mangroves, particularly which can prevent mangrove plants against oxidative damage caused by heavy metal stress (Michalak, 2006; Das et al., 2016; Rui et al., 2016; Jiang et al., 2017). 1,1-Diphenyl-2-picrylhydrazyl (DPPH) is a free radical that can remain stable at room temperature and produces a violet solution in ethanol. However, it can be reduced in the presence of an antioxidant molecule, giving rise to uncolored ethanol solutions. The use of DPPH provides a simple and rapid method for evaluating antioxidants (Mensor et al., 2001; Adjimani and Asare, 2015). Ferric-reducing antioxidant power (FRAP) is another method to estimate the antioxidant capacity of phenolic acids (Afroz et al., 2016).

To date, nutrient supply has been an effective method to induce tolerance responses to different heavy metals in plants, such as selenium (Se), phosphorus (P) and silicon (Si) (Xie et al., 2014; Cui et al., 2017), or organic acid supplies, such as salicylic acid (SA) and jasmonic acid (JA). They act against stress by enhancing antioxidant activity or chelating with heavy metals that stimulate plant growth (Irtelli and Navari-Izzo, 2006; Khan et al., 2016; Liu et al., 2016). However, there are few studies on the effects of heavy metal interactions on phenolic acid metabolism in plants. Therefore, the purpose of this study was to explore the following questions: whether the addition of ZnSO_4_ could alleviate the toxicity of Cd on plants, and whether its resistance could be attributed to heavy metal interaction effects of phenolic acid metabolism in *K. obovata*.

## Materials and methods

### Plant material collection and treatments

In this study, mature *K. obovata* hypocotyls were obtained from Jiulongjiang Estuary, Fujian Province, China. The hypocotyls of *K. obovata* were soaked in mass concentration of 1 % KMnO_4_ solution for 24 h, and then rinsed with distilled water. Hypocotyls with strong vitality were selected for sand culture until four-leaf seedlings were obtained, and then seedlings of the same size were transplanted to 2L plastic buckets in soil culture.

The soil was collected from the Cd-contaminated mangrove wetland, Jiulongjiang Estuary. Sampling points were located at 24°28′ N, 117°24′ E. The background values of Cd and Zn in sediments were 3.99 and 367.54 mg·kg^–1^, respectively. The collected soil was treated with ZnSO_4_ (ZnSO_4_·7H_2_O) after debris removal. The addition of ZnSO_4_ were 0, 80, 300, and 400 mg·kg^–1^ for Zn0, Zn80, Zn300 and Zn400 treatments, respectively. The treatment without ZnSO_4_ addition (Zn0) was used as the control, then the soils were stirred well for 30 days before the seedlings were transplanted into cultivation for 60 days. Each treatment was repeated three times.

### Determination of chlorophyll content in leaves

The mature leaves with no middle veins were cut into small pieces, and 0.2 g of the fresh leaves was weighed in a mortar containing a small amount of calcium carbonate and quartz sand. Then, 5 ml of 95% ethanol was added to the mortar, and homogenized until the tissues turned white. The mixture was filtered through a funnel, and distilled water was added to the filtrate to equal 25 ml. The absorbance of the filtrate was measured at wavelengths of 645 and 663 nm. The chlorophyll content was calculated by the equation of Sae-Lee et al. (2012).

### Determination of soluble sugar in leaves

Soluble sugar was extracted from freeze-dried powder (0.1 g) of leaves with 1.5 mL sodium phosphate buffer (pH 6.8). The samples were centrifuged for 20 min at 12,000 × g, and 0.5 mL supernatant was measured and mixed with 0.5 mL phenol (5 %) and 2.5 mL concentrated sulfuric acid. The mixture was shaken well, and left at room temperature for 30 min to color. Then, the absorbance was measured with distilled water as the blank at the wavelength of 485 nm. The standard curve was drawn with sucrose (100 μg·L^–1^) as the standard solution (Falahi et al., 2018).

### Determination of Cd and Zn content

In order to determine the content of Cd^2+^ and Zn^2+^ in the leaves, 0.05 g of dried *K. obovata* leaves were digested with 5 ml nitric acid and 1 ml H_2_O_2_ for 4 h, and then diluted with 50 ml distilled water. The total content of Cd^2+^ and Zn^2+^ in the leaves was measured directly by inductively coupled plasma mass spectrometry (ICP-MS) (Agilent 7500 ICP-MS, USA) (Chen et al., 2019).

### Detection of phenolic acids by high-performance liquid chromatography coupled to triple-quadrupole mass spectrometry (HPLC-QQQ-MS)

**Figure 1:**
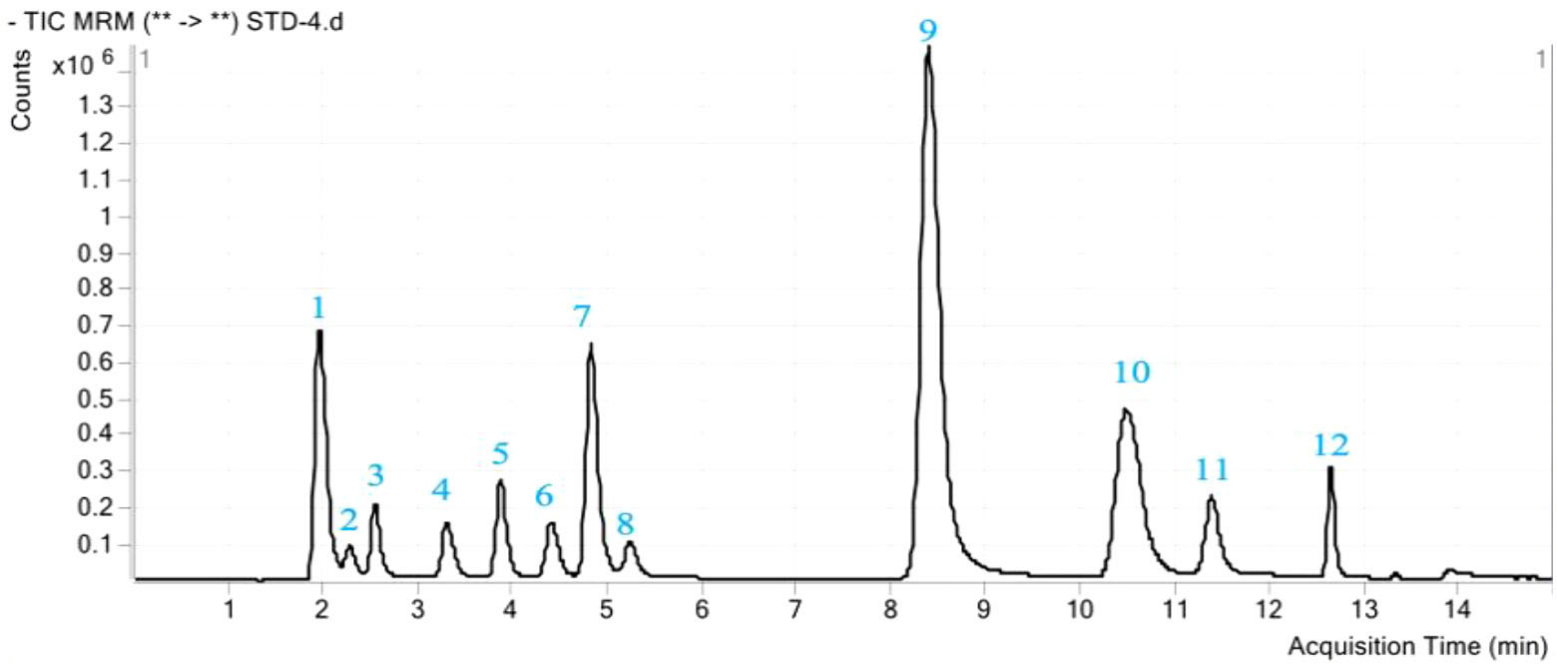
A total ion chromatogram (TIC) of phenolic acid mixture standards. The peak number represents the twelve phenolic acids as follows: 1, Pyrogallic acid; 2, Coumaric acid; 3, Protocatechuic acid; 4, Chlorogenic acid; 5, 4-Hydroxy benzoic acid; 6, Caffeic acid; 7, Syringic acid; 8, Vanillin; 9, Ferulic acid; 10, Benzoic acid; 11, Salicylic acid; 12, Cinnamic acid.

For the qualitative and quantitative determination of phenolic acids 0.1 g of ground lyophilized plant leaves were leached with 3 ml of water by oscillating at 300 rpm at 4°C for 4 h, followed by overnight maceration. The resulting extract was centrifuged at 7,500 rpm at 4°C for 10 min, and filtered with through a 0.45 μm polyvinylidene fluoride (PVDF) membrane. The Agilent 1290 LC 6490 QQQ was used for HPLC-MS analysis. Chromatographic column specifications were as follows: 00D-4462-YD, Kinetex 2.6 μ C18 100A, and New Column 100 × 3.0 mm high-performance chromatographic column. The mobile phase consisted of two solvents: (A) 2% glacial acetic acid, and (B) methanol; the gradient program started with 75% of mobile phase A at 1 min, which decreased to 73 % in 8 min and then to 50 % in 3 min and after that increased to 75 % in 2 min. The velocity of the mobile phase was 0.3 ml min^–1^, and the injection sample volume was 0.5 μl. The column temperature was 30°C, and the pressure was 500 bar. The mass spectrometer was operated in a multiple reaction monitoring mode (MRM). Mass spectrometric detection was operated in a negative ion mode after electrospray ionization. The following parameters were used for the mass spectrometer: capillary voltage, 3,371 V; drying gas (nitrogen) temperature, 350°C; flow, 11.0 L min^–1^; and collision gas, nitrogen (99.999%) (Chen et al., 2020). The *m*/*z* ratios for precursor and product ions of target analytes, as well as collision energies and retention times, are presented in **Figures 1**, **S1** (**Supplementary Materials**), and **Table 1**, respectively.

**Table 1:** LC-ESI-QQQ-MS/MS analysis of these phenolic acids.

Compound	Molecular formula	Retention time (min)	Collision energy		ESI-MS *m*/*z*	
			E1 (V)	E2 (V)	[M-H]	MS/MS fragment
Pyrogallic acid	C_6_H_6_O_3_	1.954	20	10	124.9	79.1, 97
Coumaric acid	C_6_H_4_O_4_	2.264	0	0	139.09	139.1, 95
Protocatechuic acid	C_7_H_6_O_4_	2.519	10	5	153	109.1, 153
Chlorogenic acid	C_16_H_18_O_9_	3.259	15	5	353	190.8, 352.9
4-Hydroxybenzoic acid	C_7_H_6_O_3_	3.844	10	0	137.1	93.1, 137.1
Caffeic acid	C_9_H_8_O_4_	4.418	30	30	179	134, 88.9
Syringic acid	C_9_H_10_O_5_	4.858	15	30	197.17	123, 95
Vanillin	C_8_H_8_O_3_	5.246	5	5	150.9	136.1, 151
Ferulic acid	C_10_H_10_O_4_	8.283	5	5	192.9	177.8, 134
Benzoic acid	C_7_H_6_O_2_	10.374	0	10	121	121, 77.1
Salicylic acid	C_7_H_6_O_3_	11.308	15	5	137.1	93.1, 137.1
Cinnamic acid	C_9_H_8_O_2_	12.592	5	5	147.1	103, 147

### Phenolic acid metabolism-related enzyme assay

To determine the PAL activities, 0.2 g of fresh leaves were homogenized in liquid nitrogen, and then extracted with 4 ml buffer solution (50 mM Tris pH 8.5, 14.4 mM 2-meryl ethanol, 5% w/v insoluble polyvinyl polypyrrolidone). The homogenate was centrifuged at 12,000 × g for 15 min at 4°C. Then, 0.5 mM, pH 8.0 Tris-HCl buffer and 10 μM L-phenylalanine solution were added to the obtained supernatant, and the mixture was incubated at 35 °C for 2 h. The reaction was terminated with 50 μl of 5 M hydrochloric acid. The content of reacted l-phenylalanine in the presence of PAL was determined by UV-spectrophotometry at 290 nm. The total protein concentration in the extract was measured by the Bradford (1976) method. PAL activity was expressed as nmol·min^−1^·mg^−1^ protein (Ali et al., 2006).

To determine the SKDH, CAD and PPO activities, fresh leaves were homogenized in a mortar with 50 mM potassium phosphate buffer (pH 7.0) at 4°C. The obtained homogenates were stored in an ice bath and centrifuged successively at a speed of 15,000 g for 15 min at 4°C (Kováčik et al., 2009; Blasco et al., 2013). The obtained supernatant was the crude enzyme extract. To determine SKDH activity, 0.1 M Tris-HCl buffer (pH 9), 1.45 ml of 2 mM shikimic acid, and 1.45 ml of 0.5 mM NADP was gradually added to the 0.1 mL crude enzyme solution. The absorbance was read at 340 nm over 1 min, and the molar absorbance of 6.22 mM^–1^ cm^–1^ was used to calculate its activity. CAD activity was measured by using 0.1 M Tris-HCl buffer (pH 8.8). The reaction tube contained 1.45 ml of 1 mM coniferyl alcohol, 1.45 ml of 1 mM NADP and 0.1 ml of supernatant. Measurement and calculation of CAD activity were calculated the same way as SKDH activity. PPO detection experiment was carried out in a reaction centrifuge tube containing 2.85 ml of 50 mM (pH 7.0) potassium phosphate buffer, 50 μl of 60 mM catechol and 0.1 ml of crude enzyme extract. The absorbance was read at 420 nm after 2 min. Activity was expressed as units of activity (UA) in which one unit of PPO was defined as the change in one unit of absorbance min^–1^ protein content (UA mg^–1^g^–1^ FW and recalculated with the known protein) (Kováčik et al., 2009).

### DPPH (1,1-diphenyl-2-picrylhydrazyl) free radical-scavenging activity and ferric-reducing antioxidant power (FRAP) of phenolic acids in leaves assay

A total of 3 ml of distilled water was added to 0.1 g of ground freeze-dried leaves. Then, the homogenate was shaken at 300 rpm at 4°C for 4 h, and subsequently centrifuged at 7,500 rpm at 4°C for 10 min. The supernatant was filtered with a 0.22 μm membrane to obtain the phenolic acid crude extract of the leaves.

A 0.1 g·L^–1^ DPPH solution was made by dissolving in a small amount of toluene and then diluting with 60% ethanol. Two milliliters of phenolic acid crude extract was transferred to 2 ml of 0.1 g·L^–1^ DPPH solution, and then rapidly mixed and stored at room temperature for 20 min without light. The absorbance was measured at 517 nm with 60% ethanol as a blank. The DPPH radical scavenging activity was then calculated (Afroz et al., 2016). The preparation method of the FRAP reagent was the same methods as described by Afroz et al. (2016). The above phenolic acid crude extract of 200 μl was mixed with 1.5 ml of FRAP reagent and reacted at 37°C for 4 min. The absorbance of the mixture was measured at 593 nm using distilled water as the blank. Then, the FRAP of phenolic acids in the leaves was calculated.

### Statistical analysis

All data related to biomass, Cd, Zn and phenolic acid content and enzyme activity of the *K. obovata* leaves were submitted to SPSS 25 software for variance analysis (ANOVA), the results were expressed as the Mean ± SD, Pearson correlation analysis was used for detecting relationship among the index of biomass, Cd content, Zn content, soluble sugar content, chlorophyll content, DPPH, FRAP, PAL ability, SKDH ability, CAD ability and PPO ability. Duncan multi-range test was conducted at the significance level (P ≤ 0.05) of 5 % to compare the differences among them.

## Results

### Effects of ZnSO_4_ treatments on the biomass and heavy metal content of Cd-contaminated *K. obovata* leaves

As shown in **Table 2**, the addition of ZnSO_4_ treatments significantly affected the leaves biomass, Cd content and Zn content of Cd-contaminated *K. obovata*. In contrast to the control (Zn0 treatment), the leaves biomass increased 13.7 % under Zn80 treatment, but the results significantly decreased by 15.1 and 33.4 % under Zn300 and Zn400 treatments, respectively. At the meantime, the Cd content in the leaves significantly reduced by 31.9 % under Zn80 treatment, but the results significantly increased by 74.3 and 72.5 % under Zn300 and Zn400 treatments, respectively (**Table 2**). Zn content in the leaves increased by 11.3 % under Zn80 treatment, but the results significantly increased by 21.3 and 26.7 % under Zn300 and Zn400 treatments, respectively (**Table 2**).

**Table 2:** Effects of ZnSO_4_ treatments on biomass, cadmium content and zinc content in the leaves of cadmium contaminated *K. obovata*.

Treatments	Biomass (g·plant^–1^)	Cd content (mg·kg^–1^)	Zn content (mg·kg^–1^)
Zn0	1.820 ± 0.170 c	0.113 ± 0.015 b	90.260 ± 4.860 a
Zn80	2.070 ± 0.163 c	0.077 ± 0.006 a	100.450 ± 2.234 ab
Zn300	1.545 ± 0.105 b	0.197 ± 0.003 c	109.339 ± 3.638 bc
Zn400	1.213 ± 0.094 a	0.195 ± 0.005 c	114.447 ± 10.229 c
*p*-value	***	***	**

Values are the mean ± SD (n = 3). Means followed by the same letter do not differ significantly. Levels of significance: **p < 0.01; ***p < 0.001.

### Effects of ZnSO_4_ treatments on the contents of total chlorophyll (ChlT) and soluble sugar in the leaves of Cd-contaminated *K. obovata*

**Figure 2:**
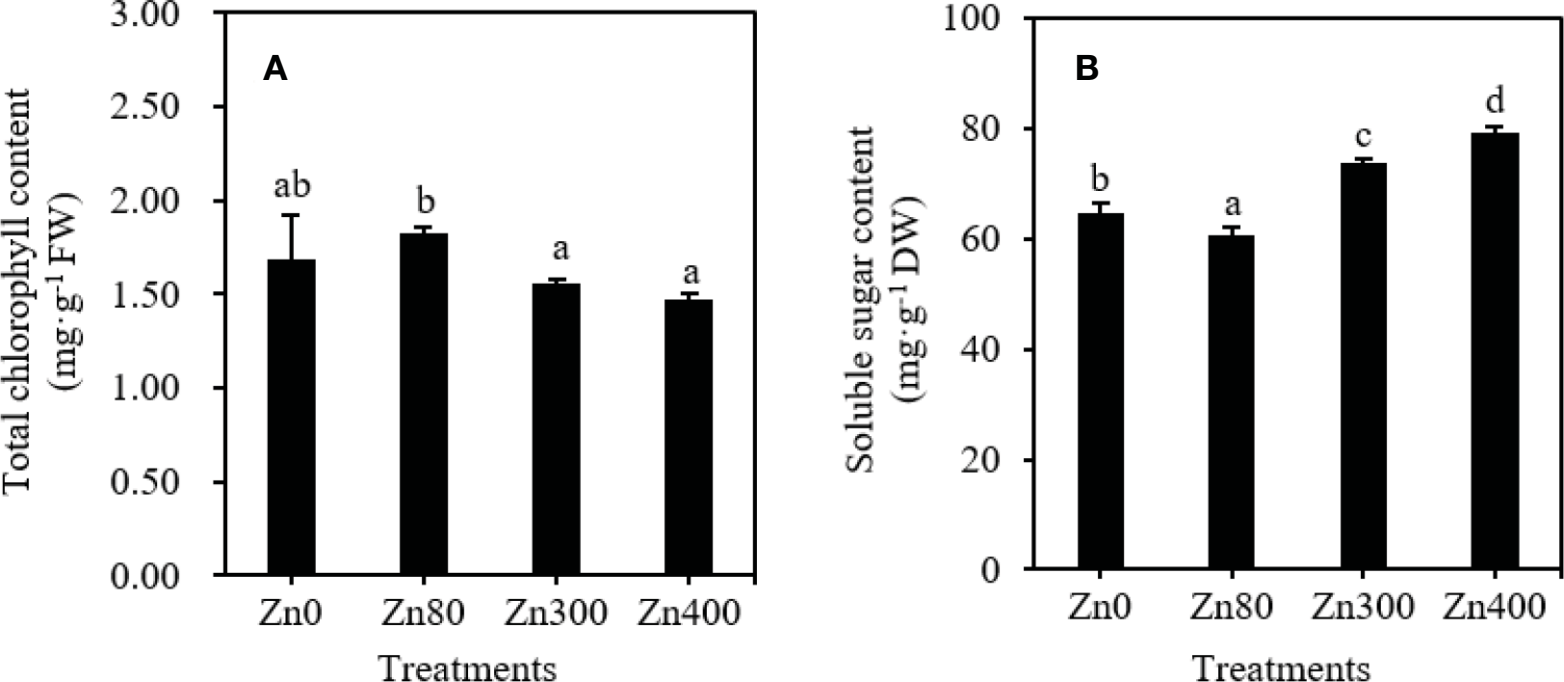
The contents of total chlorophyll **(A)**, soluble sugar **(B)** in leaves of Cd-contaminated *K. obovata* under different concentration of Zn treatments. The data are shown as the means ± SD (*n=3*).

As shown in **Figure 2A**, the highest total chlorophyll content in the leaves (1.825 mg·g^–1^ FW) was observed at Zn80 treatment, which was higher than that of Zn300 or Zn400 treatments, but no significant difference was found between Zn80 and Zn0, Zn300 and Zn400 treatments. By comparing with the control (64.80 mg·g^–1^), the soluble sugar contents under Zn300 and Zn400 treatments significantly increased by 13.5 and 22.2 %, respectively, but it significantly decreased by 6.7 % under Zn80 treatment (**Figure 2B**).

### Effects of ZnSO_4_ treatments on phenolic compounds content in the leaves of Cd-contaminated *K. obovata*

As shown in **Table 3**, the contents of phenolic compounds (not including Sal) obviously increased under Zn300 and Zn400 treatments, but no significant differences were found between Zn80 and Zn0 treatments. The maximum contents of Gal, Pro, Sal, Ben, Fer, Cou and Chl were found in Zn300 treatment, and the contents of Gal, Pro, Ben, Fer, Cou and Chl were significantly higher than that of Zn400 treatment. The maximum contents of Hyd and Cin were found in Zn400 treatment, but there were no significant differences between Zn300 and Zn400 treatments (**Table 3**). It is worth noting that, among all detected phenolic acids, Hyd, Cin and Chl were the main phenolic compounds in the levels of treated plants (**Table 3** and **Figure S2**).

**Table 3:** Effects of ZnSO_4_ treatments on phenolic compounds (PCs) in Cd-contaminated *K. obovata*.

PCs (µg·g^–1^)	Treatment				*p*-value
	Zn0	Zn80	Zn300	Zn400	
Pyrogallic acid	0.015 ± 0.003 a	0.013 ± 0.002 a	0.151 ± 0.027 b	0.038 ± 0.010 a	***
4-Hydroxybenzoic acid	1.359 ± 0.130 a	0.988 ± 0.291a	3.633 ± 1.392 b	4.180 ± 0.727 b	***
Protocatechuic acid	0.033 ± 0.006 a	0.030 ± 0.002 a	0.076 ± 0.008 b	0.035 ± 0.016 a	*
Salicylic acid	0.027 ± 0.010 a	0.022 ± 0.003 a	0.079 ± 0.030 b	0.054 ± 0.032 ab	ns
Benzoic acid	0.068 ± 0.005 a	0.073 ± 0.004 a	0.222 ± 0.037 c	0.118 ± 0.016 b	***
Ferulic acid	0.089 ± 0.017 a	0.108 ± 0.025 a	0.204 ± 0.025 b	0.134 ± 0.050 a	***
Couramic acid	0.022 ± 0.009 a	0.016 ± 0.001 a	0.415 ± 0.008 c	0.158 ± 0.074 b	*
Chlorogenic acid	2.007 ± 0.072 a	1.847 ± 0.133 a	6.228 ± 1.854 b	1.696 ± 1.974 a	***
Cinnamic acid	0.601 ± 0.045 a	0.668 ± 0.223 a	2.589 ± 0.455 b	2.785 ± 0.374 b	***

Values are the mean ± SD (n = 3). Means followed by the same letter do not differ significantly. Levels of significance: *, P < 0.05; ***, P < 0.001; ns, not significant. Gal, pyrogallic acid; Hyd, 4-hydroxy benzoic acid; Pro, protocatechuic acid; Sal, salicylic acid; Ben, benzoic acid; Fer, ferulic acid; Cou, coumaric acid; Chl, chlorogenic acid; Cin, cinnamic acid.

### Effects of ZnSO_4_ treatments on phenolic acid metabolism related enzyme activities in the leaves of Cd-contaminated *K. obovata*

The activities of phenolic acid metabolism related enzymes were obviously impacted by the addition of ZnSO_4_ treatments (**Table 4**). Compared with the control, PAL activity in leaves of the plants significantly increased by 22.4% and 43.4%, under Zn300 and Zn400 treatments. However, it declined by 27.7 % under Zn80 treatment (**Table 4**). The activities of SKDH, CAD and PPO in leaves of the plant totally increased with the levels of ZnSO_4_ treatments, and the strongest activity were detected under Zn400 treatment, which were by 86.2, 38.8 and 97.2 % higher than those of the control, respectively. But there were no differences in the activities of SKDH, CAD and PPO between Zn80 treatment and the control (**Table 4**).

**Table 4:** Effects of ZnSO_4_Effects of ZnSO4 treatments on phenolic compounds metabolism related enzymes activity in the leaves of Cd-contaminated *K. obovata*.

Activity	Treatments				*p*-value
	Zn0	Zn80	Zn300	Zn400	
PAL (nmol·min^–1^·mg^–1^)	20.30 ± 3.16 b	14.67 ± 0.47 a	24.84 ± 0.71 c	29.11 ± 1.33 d	***
SKDH (nmol·min^–1^·mg^–1^)	215.24 ± 4.333a	226.79 ± 4.60 a	325.24 ± 13.29 b	400.79 ± 18.44 c	***
CAD (nmol·min^–1^·mg^–1^)	223.82 ± 6.14 a	230.55 ± 16.39 a	294.49 ± 13.82 b	310.69 ± 9.29 b	***
PPO (UA·mg^–1^)	1.44 ± 0.06 a	1.50 ± 0.05 a	2.44 ± 0.12 b	2.84 ± 0.09 c	***

Values are the mean ± SD (n = 3). Means followed by the same letter do not differ significantly. Levels of significance: ***, P < 0.001. PAL, L- phenylalanine ammonia-lyase; SKDH, shikimic acid dehydrogenase; CAD, cinnamyl alcohol dehydrogenase; PPO, polyphenol oxidase.

### Effects of ZnSO_4_ treatment on antioxidant activity of Cd-contaminated *K. obovata* leaves

**Figure 3:**
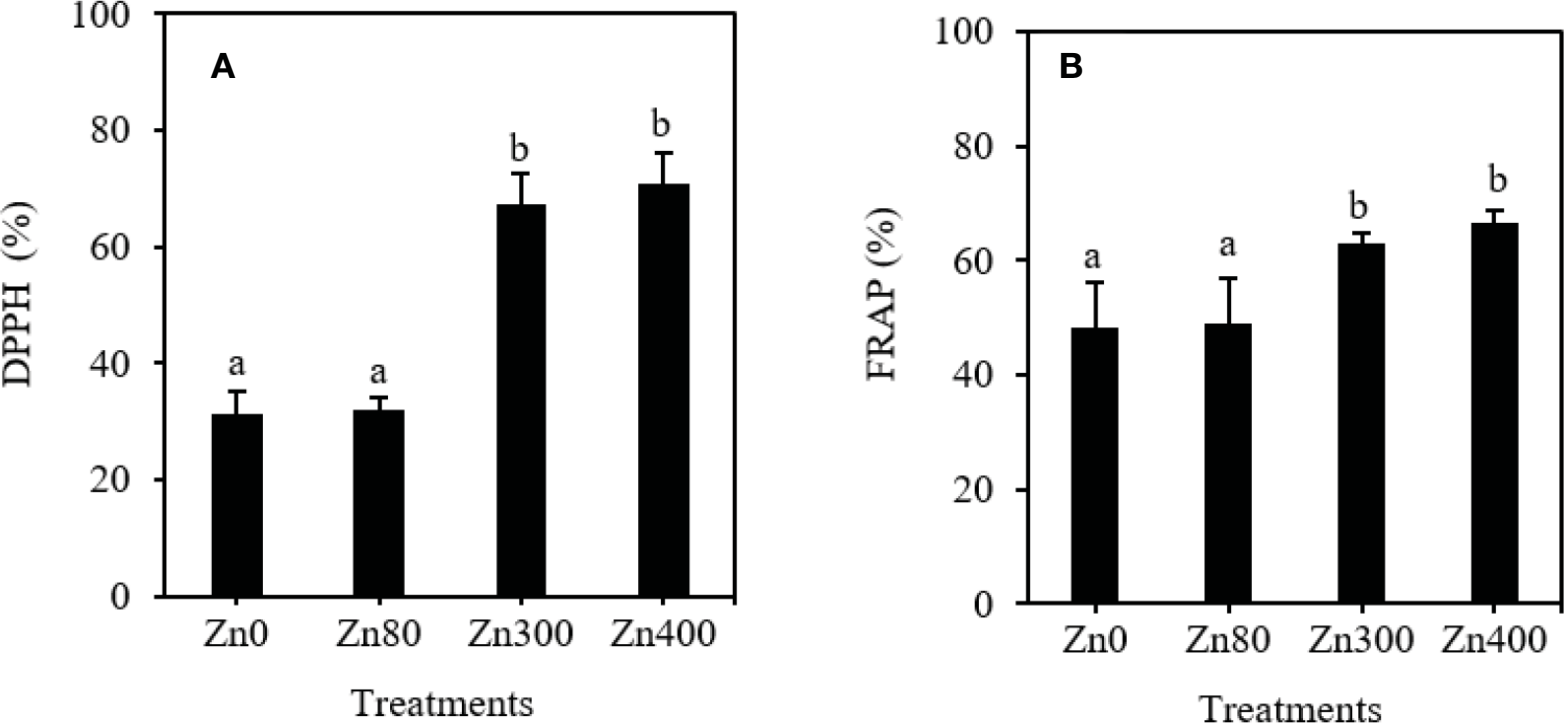
The scavenging ability of DPPH **(A)** and FRAP **(B)** of phenolic acids in leaves of Cdcontaminated *K. obovata* under different concentration of Zn treatments. The data are shown as the means ± SD (*n*=3). DPPH, 1,1-diphenyl-2-picrylhydrazyl; FRAP, ferric-reducing antioxidant power.

As shown in **Figure 3**, the DPPH free radical scavenging activity and FRAP values remarkably increased by Zn300 and Zn400 treatments. However, there were no significant differences were observed between Zn80 treatment and the control (**Figures 3A, B**). With respect to the control, the DPPH free radical scavenging activity increased by 115.6~127.3 %, and the FRAP values increased by 31.1~38.7 % under Zn300 and Zn400 treatments (**Figure 3A, B**).

### Correlations among the physiological index in the leaves

As shown in **Table 5**, the leaves biomass had a positive correlation with chlorophyll content (*r* = 0.74), but showed a negative correlation with soluble sugar content (*r* = –0.90), Cd content (*r* = –0.89) Zn content (*r* = –0.61), SKDH activity (*r* = –0.88), CAD activity (*r* = –0.83) and PPO activity (*r* = –0.87). The positive correlations were found between Zn content and soluble sugar content (*r* = 0.94), the activities of SKDH (*r* = 0.78), CAD (r = 0.80), PPO (*r* = 0.80), DPPH (*r* = 0.79) and FRAP (*r* = 0.64) in leaves. Cd contents in leaves were highly positively correlated to Zn content (*r* = 0.66), soluble sugar content (*r* = 0.94), the activities of DPPH (*r* = 0.95), FRAP (*r* = 0.86), PAL (r = 0.90), SKDH (r = 0.87), CAD (*r* = 0.91), and PPO (*r* = 0.92) in leaves, whereas Cd contents in leaves had a negative correlation with chlorophylls content (*r* = -0.74) in leaves (**Table 5**). Thus, we can see that Cd treatments inhibited the growth of *K. obovata*, while Zn application improved the free radical-scavenging ability of *K. obovata.*

**Table 5:** Pearson correlation coefficients among biomass, the contents of Cd, Zn, soluble sugar, chlorophyll and the activities of DPPH, FRAP, PAL, SKDH, CAD and PPO in the leaves of Cd-contaminated *Kandelia obovata*.

Physiological index	Cd content	Zn content	Soluble sugar content	Chlorophyll content	DPPH	FRAP	PAL activity	SKDH activity	CAD activity	PPO activity
Biomass	–0.89**	–0.61*	–0.90**	0.74**	–0.86**	–0.76**	–0.88**	–0.88**	–0.83**	–0.87**
Cd content	1	0.66*	0.94**	–0.74**	0.95**	0.86**	0.90**	0.87**	0.91**	0.92**
Zn content		1	0.70*	–0.56	0.79**	0.64*	0.57	0.78**	0.80**	0.80**
Soluble sugar content			1	–0.72**	0.93**	0.87**	0.96**	0.95**	0.91**	0.95**
Chlorophyll content				1	–0.71*	–0.55	–0.67*	–0.72**	–0.74**	–0.76**
DPPH					1	0.90**	0.84**	0.94**	0.96**	0.97**
FRAP						1	0.73**	0.84**	0.89**	0.87**
PAL activity							1	0.88**	0.82**	0.87**
SKDH activity								1	0.93**	0.98**
CAD activity									1	0.97**

*Correlation is less than or equal to 0.05 level (2-tailed); **Correlation is less than or equal to 0.01 level (2-tailed).

## Discussion

### Biomass, Cd and Zn content of *K. obovata* leaves

Biomass is one of the important indicators that reflect whether the growth of a plant is restrained (Kováčik and Bačkor, 2007; Wang et al., 2016). The addition of 300 or 400 mg·kg^–1^ ZnSO_4_ inhibited the growth of *K. obovata*, while 80 mg·kg^–1^ ZnSO_4_ promoted the growth of *K. obovata* (**Table 2**). These results suggest that treatment with this concentration of Zn^2+^ (≤ 80 mg·kg^–1^ ZnSO_4_) may initiate a special heavy metal tolerance mechanism to alleviate the Cd toxicity in *Kandelia obovata* plants to some extent.

The toxicity of heavy metal stress to plants not only causes plant growth to slow down, but also leads to the accumulation of heavy metals in plant leaves (Armas et al., 2014). The accumulation of Cd^2+^ and Zn^2+^ ions in plant leaves can trigger membrane lipid peroxidation (Jia et al., 2017), causing the production of reactive oxygen species (ROS) (Weng et al., 2012). In this study, the control plants showed lower ion concentrations of Cd^2+^ and Zn^2+^ than those in plants treated with 300 or 400 mg·kg^–1^ ZnSO_4_ (**Table 2**). The contents of Cd^2+^ and Zn^2+^ in leaves treated with high concentrations of ZnSO_4_ could account for the depress *K. obovata* leaf biomass found in these treatments (**Table 2**). In addition, 300 or 400 mg·kg^–1^ ZnSO_4_ caused an increase in the Cd^2+^ and Zn^2+^ concentrations compared to the control treatment (**Table 2**), probably because of the synergism between the Cd^2+^ and Zn^2+^. However, 80 mg·kg^–1^ ZnSO_4_ treatment inhibited the Cd^2+^ absorption in leaves (**Table 2**), likely due to the antagonism between the Cd^2+^ and Zn^2+^. Therefore, it can be seen that Cd and Zn have antagonistic effects under low concentration Zn treatment and synergistic effects under high concentration Zn treatment. The past report also confirmed these findings (Wang et al., 2016). This phenomenon could be explained by the competition between Zn and Cd because of their similar chemical properties (Wang et al., 2016). Zn is an essential element for plant growth at low concentrations, whereas Cd is a non-essential metal for plants with a low toxicity threshold (5–10 mg·kg^–1^) (Wang et al., 2016). To counteract the toxicity of Cd, plants must increase the transport of Zn from roots to shoots. Another group of authors have suggested that the interaction between Cd and Zn is synergistic. In heavy metal hyper accumulative plants, the accumulation of Zn in the stems was positively correlated with the Cd levels in plants, and the author believes that the stimulation of Cd accumulation in stems was related to the increased xylem transport of Cd from roots to shoots (Wang et al., 2016). Additionally, it was reported that most accumulated Zn could be bonding to polygalacturonic acids and carbohydrates in the cell wall (Xing et al., 2008). It has been reported that another possible mechanism for *A. marina* with excessive Zn accumulation is to secrete excess Zn through leaf glands (MacFarlane and Burchett, 2000). Under Cd and Zn pollution conditions, the growth of *K. obovata* was only slightly affected by Zn300 treatment, but an obvious reduction of foliar biomass of *K. obovata* was observed in Zn400 treatment. These results imply that low concentration Zn^2+^ treatment could alleviate Cd toxicity, but high concentration Zn^2+^ treatment could increase the toxicity of heavy metals to *K. obovata*. The main mechanism of Zn^2+^ treatment alleviate the stress by Cd pollution in *K. obovata* can be explained by the improvement of antioxidant capacity induced by Zn^2+^. This can be further confirmed by the metabolism of phenolic acids and their oxidation abilities (Lavid et al., 2001; Sgherri et al., 2003; Kováčik et al., 2009).

### Chlorophyll content, soluble carbohydrate content, and their relationships with phenolic acid metabolism

Phenolic acids are synthesized from the amino acids produced by glycolysis, which is closely related to the photosynthesis of plants (Cocetta et al., 2015). Chlorophyll consists the foundation of plant photosynthesis (Li et al., 2022). The reduction of total chlorophyll content was often found in biologically stressed plants which under nutrient imbalance or oxidative stress induced by heavy metal stress (Wang et al., 2011). In present study, we found that the total chlorophyll content in leaves decreased under Zn300 and Zn400 treatments, however, increased under Zn80 treatment (**Figure 2A**). The above results showed that adding 80 mg·kg^–1^ ZnSO_4_ under high concentration of Cd pollution could enhance the photosynthesis of plants, and increase the biomass, while adding 300 or 400 mg·kg^–1^ ZnSO_4_ treaments weaken the photosynthesis of plants. Soluble sugar is one of the products of photosynthesis, the levels of soluble sugar directly influenced the biosynthesis of phenolic acids, and the accumulation of flavonoid compounds (Cocetta et al., 2015). In our study, the change trend of soluble sugar content in leaves of *K. obovata* under Zn stress was contrary to that of total chlorophyll content (**Figure 2B**). This can be comfirmed by the significant negative correlationship between total chlorophyll content and soluble sugar content, and the significant positive correlationship between soluble sugar content and phenolic acids (**Table 5**). Therefore, the soluble sugar content can indirectly reflect the amount of phenolic acid synthesis. However, we only know that the synthesis of phenolic acid is based on sugar metabolism. So, it is necessary to further explore whether the high sugar content does necessarily lead to the high metabolism of phenolic acids.

### Effects of heavy metal stress on phenolic acid metabolism

Phenolic acids are often produced when animals and plants are subjected to abiotic and biotic stresses. In previous studies, Loponen et al. (2001) found that birch leaves from contaminated areas had higher levels of several low-molecular weight phenols than birch leaves from control areas. Blasco et al. (2013) found that phenolic acids are powerful antioxidants that can scavenge free radicals and other oxidizing substances. However, little work has been done on the response of phenolic acids to heavy metals in mangrove plants. Some studies have suggested that high concentrations of Zn and/or Cd promote phenolic acid synthesis in herbal plants (Mongkhonsin et al., 2016). Higher levels of phenolic compounds were found in both young and old leaves of *Matricaria chamomilla* following long-term exposure to 60 and 120 μM CdCl_2_ (Kováčik et al., 2009). Similar results were found in blueberry plantlets in response to Cd pollution (Manquian-Cerda et al., 2016). Whereas, whether low concentration of Zn treatment alleviating Cd toxicity is due to the upregulated metabolism of some phenolic compounds, it needed to be uncovered. This study found that long-term exposure to Zn300 or Zn400 treatments obviously increased the content of some phenolic acid metabolites (**Table 3**), but the effects of 80 mg·kg^–1^ ZnSO_4_ treatment was not as significant as those treated with 300 or 400 mg·kg^–1^ ZnSO_4_. This discrepancy in findings may be due to the heavy metal endurance of plant (Sgherri et al., 2003) and the higher concentrations of Cd (3.99 mg·kg^–1^), and Zn (367.54 mg·kg^–1^) in soil sediments of this experiment. Additionally, previous results have shown that inhibiting phenolic acid metabolism would result in early death of leaves of transgenic tobacco plant and change its cell morphology (Lodovico et al., 1998). Therefore, most of the studies have shown that phenolic acid metabolism is closely related to the normal growth of plants. Indeed, soluble sugar is formed by the photosynthesis of plants, which is closely linked to shikimic acid pathway and directly influences the amount of phenolic compound synthesis (Tato et al., 2013). So the same change trends of phenolic acid content and soluble sugar content in the leaves of Cd-contaminated *K. obovata* under different ZnSO_4_ treatments. It can be concluded that the higher the soluble sugar content in the leaves, the more phenolic acid synthesis occurs.

### Mechanism of heavy metal tolerance involved in phenolic acid metabolism

In our work, the application of ZnSO_4_ treatment in Cd-contaminated sediment, especially 300 and 400 mg·kg^–1^ ZnSO_4_ treatments, raised the content of phenolic acids in leaves of *K. obovata* (**Table 3**). The enhancement of phenolic acid content caused by ZnSO_4_ addition allowed plants to survive high concentrations of Cd stress (**Tables 2**, **3**). These phenolic acids are considered to play an important role in protecting the *K. obovata* from oxidative damage caused by Cd stress. Because phenolic acids are strong antioxidants, they can scavenge free radicals and ROS (Kim et al., 2006). The properties of phenolic acids are determined by their structures, which mainly depend on the number and position of the hydroxyl groups on the aromatic ring (Kim et al., 2006). The phenolic acids in *K. obovata* plants are usually divided into two groups: benzoic and cinnamic acid derivatives. The antioxidant activity of cinnamic acid derivatives such as ferulic acid, caffeic acid and *p*-coumaric acid was higher than that of hydroxybenzoic acid derivatives such as *p*-hydroxybenzoic acid vanillic acid and salicylic acid (Kim et al., 2006). That is because that the presence of the CH=CH–COOH group in the hydroxycinnamic acids is considered to be key for the significantly higher antioxidative efficiency compared to the COOH in the hydroxybenzoic acids (Kim et al., 2006). In addition, phenolic acids can mitigate the effects of oxidative stress due to its electron donor functions and can be used as an excellent substrate for some antioxidant enzymes (Blasco et al., 2013). **Table 3** shows that the application of ZnSO_4_ significantly induces the biosynthesis of cinnamic acid and benzoic acid, which can scavenge free radicals efficiently and stimulate antioxidant activity under different types of adverse stress in plants (Blasco et al., 2013), and the 300 and 400 mg·kg^–1^ ZnSO_4_ treatments can stimulate the synthesis of benzoic and cinnamic acid derivatives more effectively than the 80 mg·kg^–1^ ZnSO_4_ treatment. Therefore, under the same concentration of Cd stress, plants treated with ZnSO_4_ at low concentration are more resistant to Cd than those without ZnSO_4_. This is because zinc ion can enhance the plant's tolerance to heavy metals by stimulating the metabolism of phenolic acid. The Zn supplementation could not only be inhibitory for Cd-induced oxidative stress (Garg and Kaur, 2013) by inducing the biosynthesis of phenolic compounds (Mongkhonsin et al., 2016), but also be used as a remediation agent for heavy metals (Rai et al., 2019). The plant phytoavailability of Cd (≥ 54.13%) was significantly reduced in the soil mixed with single superphosphate, triple superphosphate, and calcium magnesium phosphate sepiolite in conjunction with ZnSO_4_ (Guo et al., 2017). All of these studies indicated that ZnSO_4_ can alleviate Cd stress and ZnSO_4_ is an effective remediation agent for Cd pollution soil.

It was reported that the activities of PAL, SKDH, CAD and PPO were enhanced for plants to better resist Cd pollution (Irtelli and Navari-Izzo, 2006; Kováčik et al., 2009). Similar results can be found in our work (**Table 4**). PAL mediates phenylalanine to produce cinnamic acid, which is a key branching point in primary and secondary metabolism, and it is also the first and most important regulatory step in the formation of many phenolic acids (Cocetta et al., 2015). SKDH is a member of shikimate pathway which can convert simple carbohydrates into aromatic amino acids including phenylalanine (Kováčik et al., 2009). It is one of the enzymes that controls the reaction of carbohydrates toward to phenolic acids and provides a substrate for PAL (Blasco et al., 2013; Chen et al., 2019). Plants can generate gallic acid and 4-hydroxybenzoate through shikimate pathways (Zhou et al., 2019). The 4-hydroxybenzoate and 3,4-dihydroxybenzoic acid are produced by general phenylpropanoid pathway with cinnamic acid precursor. Cinnamic acid is also a precursor for the synthesis of benzoic acid, salicylic acid, caffeic acid, ferulic acid, coumaric acid and vanillin (Renault et al., 2017; Barros et al., 2019). Cinnamic acid and *p*-coumaric acid are substrates of CAD (Barros et al., 2019), which provide precursors for the biosynthesis of lignin (Chen et al., 2019). In fact, phenolic compounds are the substrates of enzymatic browning reactions, which can form colored quinones under the enzymatic oxidation by PPO (Léchaudel et al., 2018). However, the high level of PPO activity reduces the free oxygen level available for ROS production, which may be one of the reason for the reduced ROS level of cellular under stress conditions (Léchaudel et al., 2018). As can be seen from **Table 4**, the lowest activities of the SKDH, CAD, and PPO found in the control plants (**Table 4**), corresponding to the minimum concentrations of phenolic acids in *K. obovata* plants. On the contrary, the application of 300 or 400 mg·kg^–1^ ZnSO_4_ treatment increased the activity of PAL, SKDH, CAD and PPO compared with the 80 mg·kg^–1^ ZnSO_4_ treatment (**Table 4**). These findings could explain why the concentration of phenolic acids increased (**Table 3**), why phenolic acids can protect plants against heavy metal stress, and why the leaf biomass of the 80 mg·kg^–1^ ZnSO_4_ treatment group was higher than that of the 300 and 400 mg·kg^–1^ ZnSO_4_ treatments groups. It has been reported that stronger phenolic acid metabolism related enzyme activities and the content of phenolic acids are linked to the resistance of plants to abiotic stress (Blasco et al., 2013). Zn^2+^ treatment contributed to the increase in SKDH enzyme activity, which may be due to the promoting influence of Zn on photosynthesis and carbohydrate synthesis. And the additional carbohydrates can be provided to meet the increased synthesis of phenolic acids under Cd stress. PPO activity is correlated with the production of quinine and ROS, so the increase in PPO activity will aggravate oxidative stress (Blasco et al., 2013). The results showed that *K. obovata* had the lowest foliar biomass under the 400 mg·kg^–1^ ZnSO_4_ treatment (**Table 2**), but its PPO activity was the highest (**Table 4**), which is related to the greater oxidative stress and the formation of more ROS in these plants. While, the application of 80 mg·kg^–1^ ZnSO_4_ inhibited PPO activity (**Table 4**), the reduced PPO activity would decrease the concentration of H_2_O_2_ in the plant, and thus, enhance the plant’s ability to resist stress (Thipyapong et al., 2004).

### Free radical scavenging ability of phenolic acids

DPPH and FRAP are important indexes to measure the antioxidant potential of phenolic acid effectively (Cocetta et al., 2015). The DPPH and FRAP values in plant leaves increased under ZnSO_4_ treatment; adding 300 or 400 mg·kg^–1^ ZnSO_4_ increased the antioxidant capacity of phenolic acid extracts from leaves. In addition, some phenolic acid content of plants under high concentration ZnSO_4_ treatment was also higher than that of under low concentration ZnSO_4_ treatment (**Table 3**). Therefore, the higher the content of some phenolic acids in leaves, the stronger the antioxidant capacity of leaves and the stronger the resistance to Cd oxidative damage. It can also be seen from the Pearson correlation values in **Table 5** that the content of Cd and Zn in leaves is highly positively correlated with the activity of enzymes related to phenolic acid metabolism and the content of chlorophyll is highly negatively correlated with the activity of those enzymes; however, the soluble sugar content and the antioxidant capacity were highly positively correlated with the activity of those enzymes. These results indicated that under the pollution of Cd and Zn, *K. obovata* can stimulate the metabolism of phenolic compounds, sugar metabolism, and photosynthesis related to phenolic acid metabolism to improve the antioxidant capacity of plants to resist the oxidative damage caused by heavy metal stress; 80 mg·kg^–1^ ZnSO_4_ can alleviate Cd toxicity by improving the ability of phenolic acid metabolism of plants.

## Conclusion

Our findings showed that the application of ZnSO_4_ to Cd-contaminated sediment increased phenolic acid contents and related enzyme activities in leaves of *K. obovata*. These increases also led to an increase of the biosynthesis of hydroxycinnamic acids, and hydroxybenzoic acid and their derivatives, which have a strong ROS scavenging ability in plant leaves, and can be used as protective compounds for mangrove plants under Cd stress conditions. Overall, this work revealed that the application of repair agents containing low concentrations of ZnSO_4_ is an effective strategy to raise the resistance of Cd contaminated mangrove plants. This is not only beneficial to improve the growth and reduce Cd accumulation of mangrove plants, but also increase the nutritional value of animal diets, including phenolic compounds the trace element of Zn, in the food chains of mangrove ecosystems.

## Data availability statement

The original contributions presented in the study are included in the article/**Supplementary Material**. Further inquiries can be directed to the corresponding author.

## Author contributions

The author confirms being the sole contributor of this work and has approved it for publication.
