# Supplementary material for: Mechanism of Zn alleviates Cd toxicity in mangrove plants (Kandelia obovata)
Source: Front Plant Sci. 2023 Feb 2;13:1035836. doi: 10.3389/fpls.2022.1035836 (PMC9932546; doi:10.3389/fpls.2022.1035836)
Supplement: Supplementary file 1 [file DataSheet_1.docx]

1– Pyrogallic acid 2– Coumaric acid 3– Protocatechuic acid

153.0

109.1

139.1

4– Chlorogenic acid 5– 4-Hydroxy benzoic acid 6– Caffeic acid

190.8

137.1

352.9

7– Syringic acid 8– Vanillin 9– Ferulic acid

151.0

136.1

134.0

10– Benzoic acid 11– Salicylic acid 12– Cinnamic acid

147.0

103.0

137.1

121.0

Figure S1 HPLC-(ESI)QQQ-MS MS spectra of chromatographic peaks obtained from phenolic acids standards. Those blue diamonds represent pseudo molecular ions of the precursor ions.

**A**


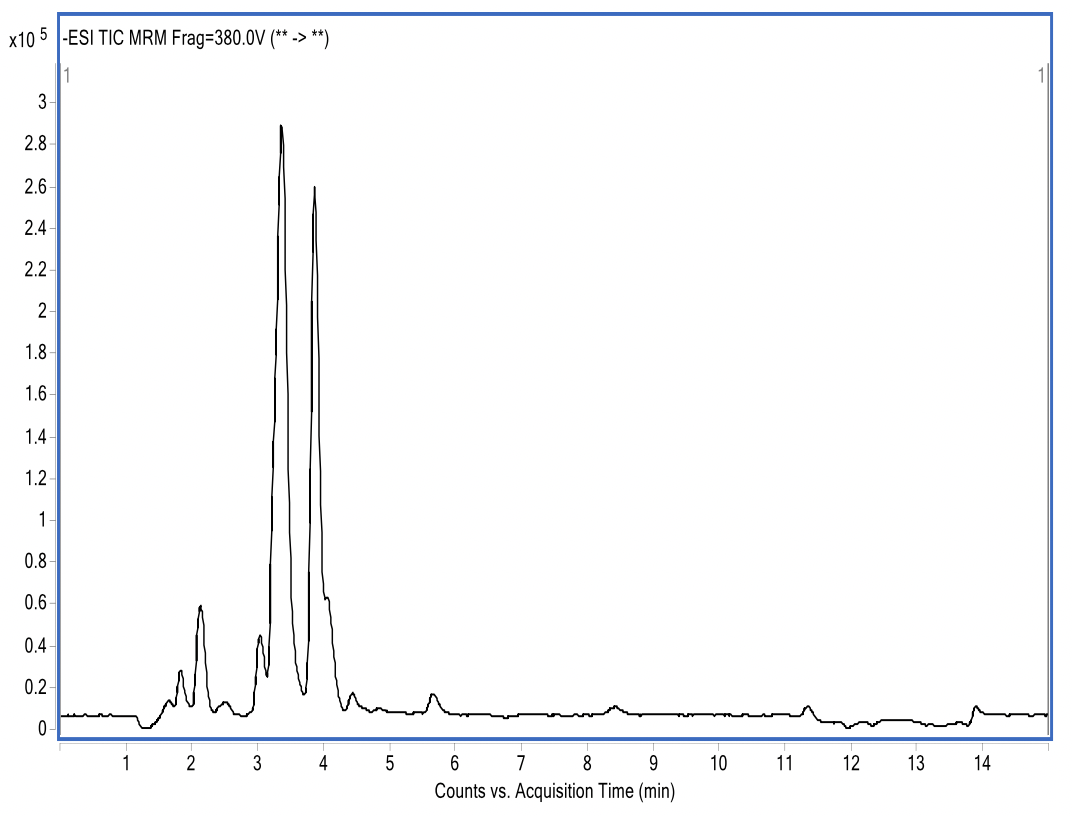


**B**


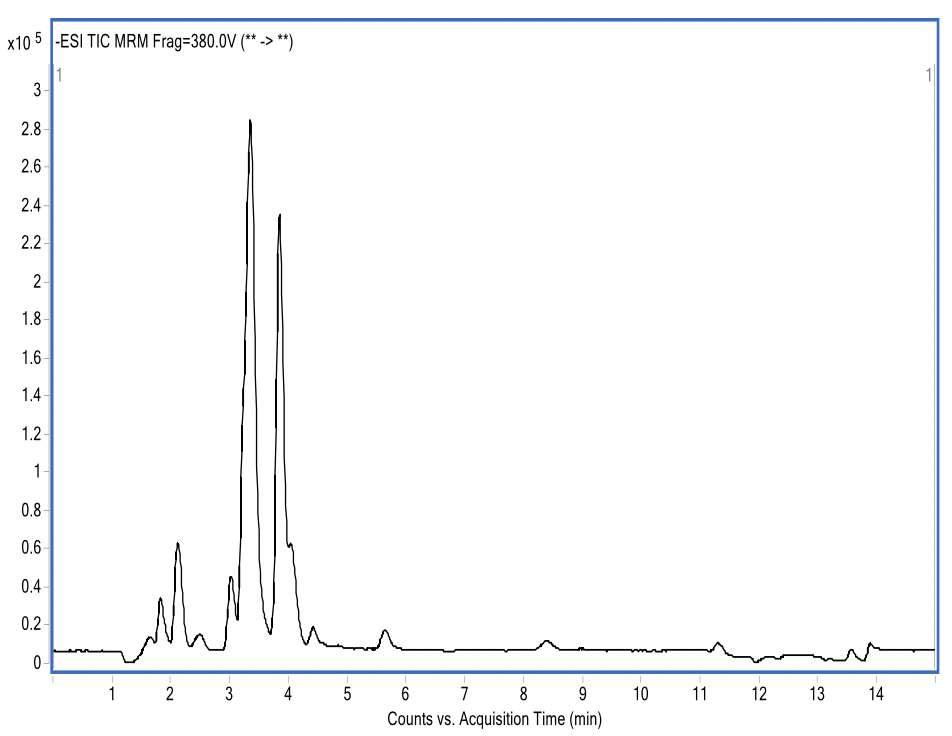


**C**


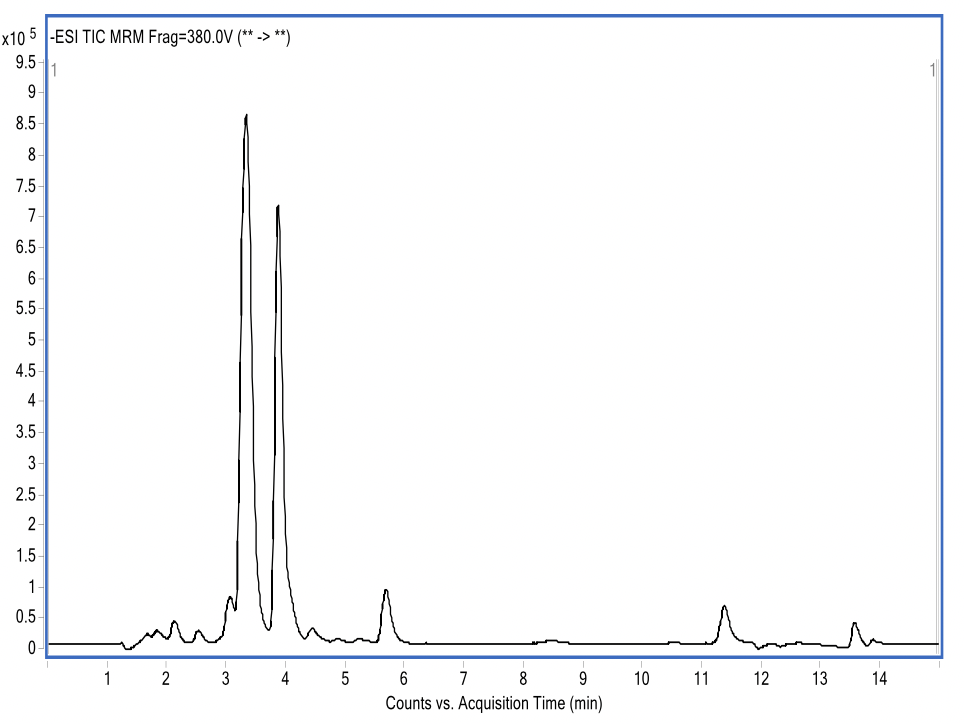


**D**


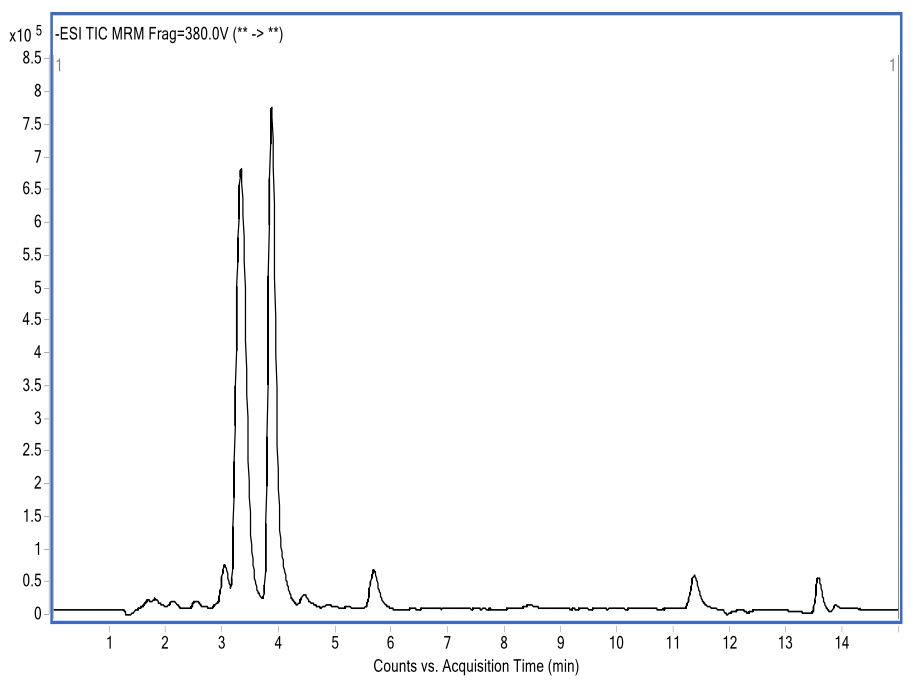


Figure S2 Ion chromatograms of cadmium-contaminated *K. obovata’* leaves under different concentration of Zn treatment. (A) control treatment; (B) 80 mg·kg^-1^ Zn treatment; (C) 300 mg·kg^-1^ Zn treatment; (D) 400 mg·kg^-1^ Zn treatment
